# Hydrogen/Deuterium Exchange Mass Spectrometry: Fundamentals, Limitations, and Opportunities

**DOI:** 10.1016/j.mcpro.2024.100853

**Published:** 2024-10-09

**Authors:** Lars Konermann, Pablo M. Scrosati

**Affiliations:** Department of Chemistry, The University of Western Ontario, London, Ontario, Canada

**Keywords:** protein folding, protein dynamics, protein aggregation, protein binding, protein mutation, protein digestion, protein modeling, protein stability, molecular dynamics simulation, electrospray ionization, mass spectrometry, isotope effect

## Abstract

Hydrogen/deuterium exchange mass spectrometry (HDX-MS) probes dynamic motions of proteins by monitoring the kinetics of backbone amide deuteration. Dynamic regions exhibit rapid HDX, while rigid segments are more protected. Current data readouts focus on qualitative comparative observations (such as “residues X to Y become more protected after protein exposure to ligand Z”). At present, it is not possible to decode HDX protection patterns in an atomistic fashion. In other words, the exact range of protein motions under a given set of conditions cannot be uncovered, leaving space for speculative interpretations. Amide back exchange is an under-appreciated problem, as the widely used (*m*–*m*_0_)/(*m*_100_–*m*_0_) correction method can distort HDX kinetic profiles. Future data analysis strategies require a better fundamental understanding of HDX events, going beyond the classical Linderstrøm-Lang model. Combined with experiments that offer enhanced spatial resolution and suppressed back exchange, it should become possible to uncover the exact range of motions exhibited by a protein under a given set of conditions. Such advances would provide a greatly improved understanding of protein behavior in health and disease.

## Highlights

• Bottom-up HDX-MS yields qualitative insights into protein dynamics.
• Amide back exchange is an under-appreciated problem.
• The (*m*–*m*_0_)/(*m*_100_–*m*_0_) back exchange correction can produce large errors.
• Future analysis strategies will have to go beyond the Linderstrøm-Lang model.
• Enhanced experimental/modeling workflows will uncover details of protein motions.

## Graphical Abstract

**Figure:**
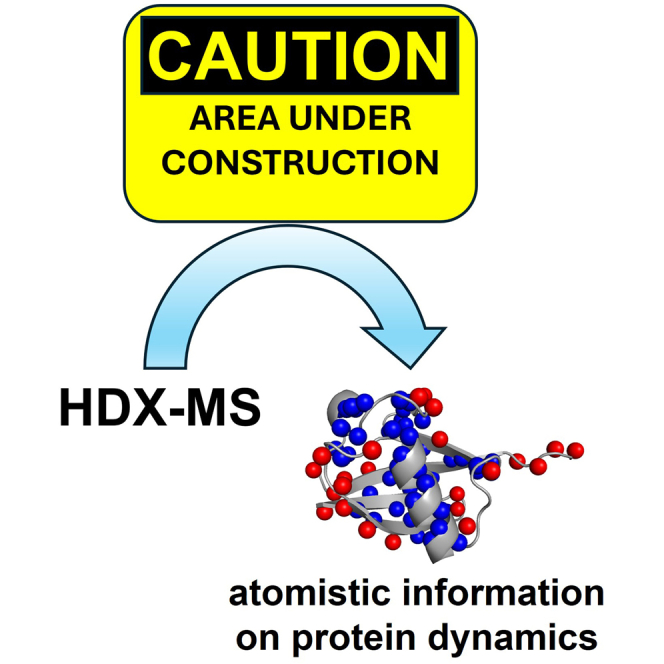

## In Brief

HDX mass spectrometry is widely used for probing protein conformational dynamics. Commonly used bottom-up HDX methods reveal qualitative changes in protection patterns in response to ligand binding or other stimuli, with moderate spatial resolution. It should be possible to develop enhanced experimental approaches and data analysis strategies that provide truly quantitative and atomistic insights into protein motions. Here we discuss some of the bottlenecks that have to be dealt with for attaining this goal.

Proteins perform countless biological functions, including catalysis (enzymes), host defense (antibodies), energy conversion (respiratory chain), and signaling (cellular receptors). Prerequisites for these functions are highly ordered tertiary and quaternary structures that are stabilized by intra- and intermolecular noncovalent contacts (1). X-ray crystallography, cryo-electron microscopy, and nuclear magnetic resonance (NMR) spectroscopy provide atomically resolved protein structures (2). In addition to their intricate conformations, a central aspect of proteins is their ability to undergo motions on a wide range of lengths and time scales, causing incessant fluctuations around the average native structure. Some of these dynamics are driven by external energy sources such as ATP hydrolysis or a transmembrane gradient (3, 4). More generally, dynamic motions arise from the thermal energy of the protein and its surrounding solvent (5, 6). Conformational dynamics are linked to protein function, *e.g.*, for enzyme catalysis (7, 8, 9, 10, 11, 12, 13), and the reversible binding of allosteric effectors (14, 15, 16, 17, 18). However, conformational dynamics also allow proteins to visit structures that are prone to aggregation (19), resulting in loss of function and causing cytotoxicity (20, 21). Thus, a detailed understanding of proteins in health and disease requires techniques that can provide detailed insights into conformational dynamics.

## Techniques for Probing Protein Dynamics

Hydrogen/deuterium exchange (HDX) mass spectrometry (MS) represents one of the most widely used techniques for interrogating protein conformational dynamics. Starting with the pioneering work of a few laboratories in the early 1990s (22, 23, 24, 25), there now is a large HDX-MS user community in industry and academia (16, 26, 27, 28). HDX-MS possesses several very attractive features, particularly compared to HDX-NMR, an older approach that has somewhat fallen out of favor (29, 30, 31). Advantages of HDX-MS compared to HDX-NMR include its accessibility, conceptual simplicity, high sensitivity, compatibility with natural isotope abundances (instead of having to express isotopically enriched constructs), and applicability to proteins of virtually unlimited size. HDX-MS can probe the individual contributions of co-existing populations in samples containing multiple conformers, while HDX-NMR only provides population-averaged data (25, 32, 33). Before discussing HDX-MS in more detail, we will briefly consider some complementary techniques for probing protein dynamics.

X-ray crystallography provides coordinates and B-factors of individual atoms. These B-factors partially reflect atomic thermal motions in the crystal. However, there are also static crystal disorder contributions (34), such that it is not clear to what extent B-factors mirror protein dynamics in solution (34, 35), even though they are frequently interpreted in this context.

Förster resonance energy transfer (FRET) spectroscopy can detect protein dynamics with single molecule sensitivity (36, 37). Unfortunately, the atomistic details obtainable from FRET are limited, because the data only report on the distance and relative orientation of protein-linked chromophore pairs. Additional insights are obtainable by examining a range of constructs that have FRET pairs in different locations (38), but this approach is laborious. Also, coupling with FRET chromophores may alter protein structures and dynamics (39).

NMR spin relaxation methods are a powerful tool for measuring dynamic fluctuations (40, 41). Initially, such experiments were limited to small monomeric systems, but the application of methyl–transverse relaxation-optimized spectroscopy (TROSY) has extended the reach of NMR dynamic measurements to large multi-protein complexes (42). The number of users employing these NMR approaches is small, likely because the need for isotopically labeled proteins (such as ^15^N backbone or ^13^C methyl) creates barriers for the adoption of this technology (43).

Molecular dynamics (MD) simulations are a computational tool that can model biomolecular behavior, including protein folding and conformational fluctuations (44). Early MD studies were restricted to very short time windows of a few nanoseconds (45), but recent hardware and software advances have pushed the envelope to milliseconds and beyond, even for large proteins in explicit water (46, 47). The Newtonian “balls on springs” approach inherent to MD simulations involves many simplifying assumptions. As a result, careful validation of MD data against experiments is essential (48).

Following this brief survey, we now return to HDX-MS. Numerous reviews have discussed HDX-MS methodology, while also highlighting a wide range of interesting applications (26, 28, 49, 50, 51, 52, 53, 54, 55, 56, 57). Instead of comprehensively re-reviewing this vast field, we will only focus on a few key issues.

## Can Proteins “Feel” the D_2_O Environment?

HDX-MS probes the deuteration of backbone NH sites as a function of time, after transferring the protein from H_2_O into D_2_O-based labeling buffer. HDX is typically performed under physiologically relevant conditions, *i.e.*, in near-neutral solution around 295 K. Most practitioners rely on the relationship pD = (pH electrode reading) + 0.4 for preparing samples with equivalent D^+^ and H^+^ activity, *e.g.*, a D_2_O solution that provides a reading of 7.0 (= pD 7.4) is considered equivalent to an H_2_O solution with a reading of 7.4 (58, 59, 60). Regardless of this pH correction, switching the solvent from H_2_O to D_2_O can affect protein stability, evident from a slightly increased melting temperature (61). This subtle stabilization may be caused by strengthened solvent-solvent contacts in D_2_O vs. H_2_O, while the stability of intramolecular NH vs. ND H-bonds is indistinguishable (60). Thus, HDX is a fairly “benign” labeling technique, unlike chemical footprinting (62) or crosslinking (63). The latter methods involve the incorporation of covalently bound moieties that can perturb protein behavior, requiring careful controls (64, 65). This is different in HDX, where the protein continues its natural dynamic motions before and after deuteration.

## Can HDX-MS Provide Detailed Atomistic Insights Into Protein Dynamics?

Although current HDX-MS methodologies are already highly useful, additional advances are hopefully going to take place in the coming years. What would be the outcome of an “ideal” HDX-MS experiment? X-ray crystallography remains the gold standard for producing static high-resolution structural data for native proteins, where each heavy atom is defined by its x/y/z coordinates within ∼1 Å (2). Ideally, HDX-MS would yield comparable information about protein *dynamics*, *i.e.*, the exact range of motions performed by each residue. Such a readout would reveal the specific conformations populated by a protein under any set of conditions. There have already been attempts to use data from HDX-MS and complementary techniques in this context (66, 67, 68, 69, 70, 71, 72, 73, 74, 75, 76). However, several challenges currently impede progress toward a quantitative and truly atomistic interpretation of HDX-MS data, as discussed in the subsequent sections.

## The Linderstrøm-Lang Model

The following considerations apply to a sample containing many copies of a native protein. Mixing with excess D_2_O defines the zero time point of the HDX measurement. All amino acids (except for Pro and the N-terminus) possess a backbone NH, providing a closely spaced series of probes along the sequence. The deuteration kinetics of residue *j* proceed with the first-order rate constant *k*_HDX_j_ during the deuteration period *t*_HDX_, such that the deuterium content *D*_j_ at this residue is

(1) $$D_{j}\left(t_{HDX}\right)=1-\exp\left(-k_{HDX\_j}t_{HDX}\right)$$

where *D*_j_(∞) = 1 corresponds to complete deuteration. Strictly speaking, the right-hand side of Equation 1 still has to be multiplied by the D_2_O mole fraction of the labeling buffer (often 0.9). For simplicity we assume that HDX takes place in pure D_2_O, such that this factor can be omitted.

Since the 1960s, *k*_HDX_j_ values have been interpreted within the Linderstrøm-Lang model (52, 77), which posits that HDX is governed by H-bond fluctuations. In this model, H-bonded backbone sites NH_j_ spend most of their time in a closed state, characterized by an intact NH···OC contact that precludes deuteration. Short-lived fluctuations cause rare transitions to an open state where the H-bond is disrupted with rate constant *k*_op_j_, before the site closes again with rate constant *k*_cl_j_. During the brief periods when NH_j_ is open, the amide undergoes deuteration with the chemical rate constant *k*_ch_j_ (Equation 2) (52).

(2) $${\text{NH}}_{j\_\text{closed}\;}\overset{k_{\text{op}\_j}}{\underset{k_{\text{cl}\_j}}{⇌}}{\text{NH}}_{j\_\text{open}\;}\overset{k_{\text{ch}\_j}}{\to }{\text{ND}}_{j}$$

Here, *k*_ch_j_ represents the rate constant that would apply if NH_j_ were permanently open (*i.e.*, under conditions where *k*_HDX_j_ = *k*_ch_j_). The value of *k*_ch_j_ depends on pD, temperature, and on the side chains on residues *j* and *j*-1. Spreadsheets for *k*_ch_j_ calculations are available from the Englander laboratory (hx2.med.upenn.edu) (59, 78), providing the basis of the calculations below.

We will focus on the commonly encountered EX2 regime, characterized by *k*_cl_j_ >> *k*_op_j_ and *k*_cl_j_ >> *k*_ch_j_. In this scenario, each NH_j_ has to undergo many closed ⇌ open fluctuations before deuteration takes place, such that

(3) $$k_{HDX\_j}=K_{op\_j}\times {k_{ch\_}}_{j}$$

where *k*_op_j_/*k*_cl_j_ = *K*_op_j_ << 1 is the equilibrium constant of the opening transition (52, 79). A large *K*_op_j_ implies fast deuteration, representing highly dynamic amides that spend a considerable fraction of time in the open state. Conversely, small *K*_op_j_ values are associated with rigid segments.

**Fig. 1:**
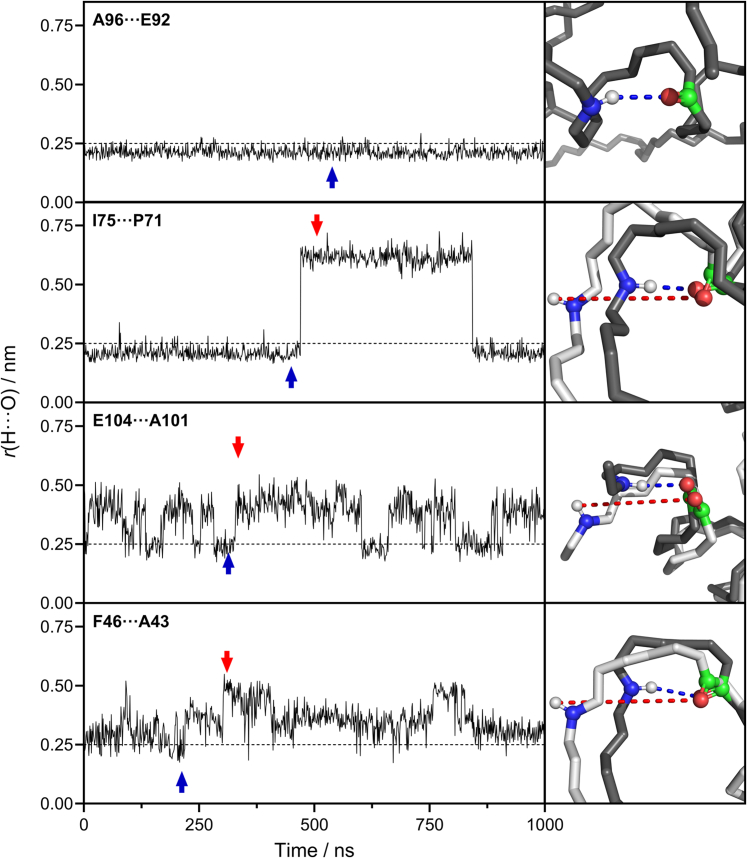
**MD simulations of amide backbone fluctuations for several NH OC contacts in cytochrome *c***. Panels on the left show hydrogen-oxygen distances *versus* time. For each pair, the NH donor is listed first. Vertical dotted lines at 0.25 nm indicate the H-bond cut-off. The top panel illustrates an H-bond that remains permanently closed during the 1 μs simulation window. All others undergo closed ⇌ open transitions. Panels on the right illustrate MD snapshots as overlays of NH_closed_ (*blue dashed*) and NH_open_ (*red dashed*) conformers, for time points indicated by the blue and red arrows. Element coloring: N (*blue*), H (*white*), O (*red*), C (*green*). Reproduced with permission from ref. (80) Copyright 2021, American Chemical Society.

The postulated closed ⇌ open fluctuations of the Linderstrøm-Lang model (52, 77), are difficult to confirm experimentally, but they are readily observable in MD simulations as illustrated in Figure 1 (80). While reassuring, such computational data do not prove the correctness of the Linderstrøm-Lang model. Several fundamental questions remain (81), such as (i) the extent to which NH···OC contacts have to open up to allow deuteration to take place. Past work related to this issue has focused on NH interactions with D_2_O (82), while in reality, the interaction with OD^-^ (which catalyzes HDX at physiological pD) is a more important aspect (59, 83). Hence, future investigations are required to determine what exactly comprises an “open” state in Equation 2. (ii) It remains contentious whether HDX kinetics are affected by the solvent accessibility of NH sites, in the absence of closed ⇌ open fluctuations (55). Some authors noted that solvent-accessible amides are protected, as long as they form intramolecular H-bonds (84) Others proposed that solvent accessibility is a major factor for determining HDX rates (74, 85). Disconcertingly, neither H-bonding nor solvent accessibility can account for the HDX behavior of a staggering 15 out of 72 NH sites in ubiquitin (86). (iii) While the Linderstrøm-Lang model exclusively focuses on H-bonding, there are alternative proposals that emphasize the role of electrostatic effects. Local electrostatics can modulate HDX rates by (de)stabilizing the -C(O^-^)=N- intermediate formed during NH → ND conversion (87, 88). Electrostatics may also alter the local concentration of OD^-^ catalyst. (iv) It is unclear whether all types of conformational dynamics are associated with H-bond opening/closing. Recent data on cytochrome *c* suggest that some fluctuations can be “HDX-silent”. In other words, there may be structural events that are only weakly coupled to changes in H-bonding, making them virtually impossible to track by HDX methods. Under such conditions, HDX-MS will provide an incomplete picture of protein dynamics (80).

The aforementioned points i - iv make it clear that despite its beautiful simplicity, the Linderstrøm-Lang model may not fully capture all intricacies of the HDX process, warranting future mechanistic investigations. Englander *et al*. noted a “*need for returning to a detailed hydrogen by hydrogen analysis to examine the bases of structure-rate relationships”* (81). Such statements from one of the leaders in the field should give reason to pause, as they imply that a proper interpretation of HDX-MS data is more difficult than commonly thought.

## Quantifying HDX Protection

When considered in isolation, *k*_HDX_j_ values provide only relatively little information, because individual NH__j_ sites can have quite different chemical exchange properties. For example, amides between two Cys, His, or Asn have *k*_ch_j_ > 40 s^−1^, whereas those between two Ile or Leu have *k*_ch_j_ ≈ 1 s^−1^ at pD 7.4 and 22° C (59, 78). Thus, *k*_HDX_j_ values can differ by more than one order of magnitude, even if the corresponding amides share the same *K*_op_j_ (Equation 3). To compensate for these chemical exchange effects, *k*_HDX_j_ values can be reported as protection factors *P*_j_ (or log *P*_j_) that reflect by how much deuteration is slowed compared to an unprotected site.

(4) $$P_{j}=\frac{k_{\text{ch}\_j}\;}{k_{\text{HDX}\_j}\;}$$

**Fig. 2:**
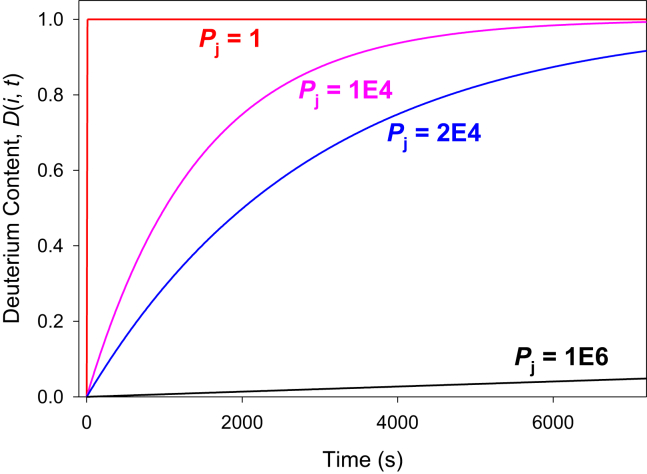
**Protection factor effects on HDX kinetics.** The deuteration kinetics of an NH_j_ site were calculated using Equation 1 for different protection factors *P*_j_ in a 2 h time window. Parameters used: *k*_ch_j_ = 6.9 s^−1^, corresponding to poly-Ala at 295 K in D_2_O solution at pD 7.4.

Equations 3 and 4 imply that, within the Linderstrøm-Lang model, *P*_j_ = 1/*K*_op_j_. Protection factors can vary greatly for different sites in the same protein, from *P*_j_ ≈ 1 (essentially unprotected, *e.g.*, in flexible loops) all the way to > 10^6^ (strongly protected, in rigid α-helices or β sheets) (29, 30). Figure 2 illustrates single-site deuteration kinetics for different *P*_j_ values.

The commonly used method for calculating *k*_ch_j_ values considers sequence, pH (or pD), and temperature. It relies on NMR-detected exchange rates measured in dipeptides that are considered to be completely unprotected (59, 78). It remains unclear whether such dipeptides adequately represent open sites in proteins, where even extensively disordered regions may still have *P*_j_ > 1 (78). Also, currently used *k*_ch_j_ values may not capture effects mediated by some additives (57, 89). These issues notwithstanding, *k*_ch_j_ values are calculated using refs. (59, 78) continue to be widely used.

## The Standard Bottom-up HDX-MS Workflow

**Fig. 3:**
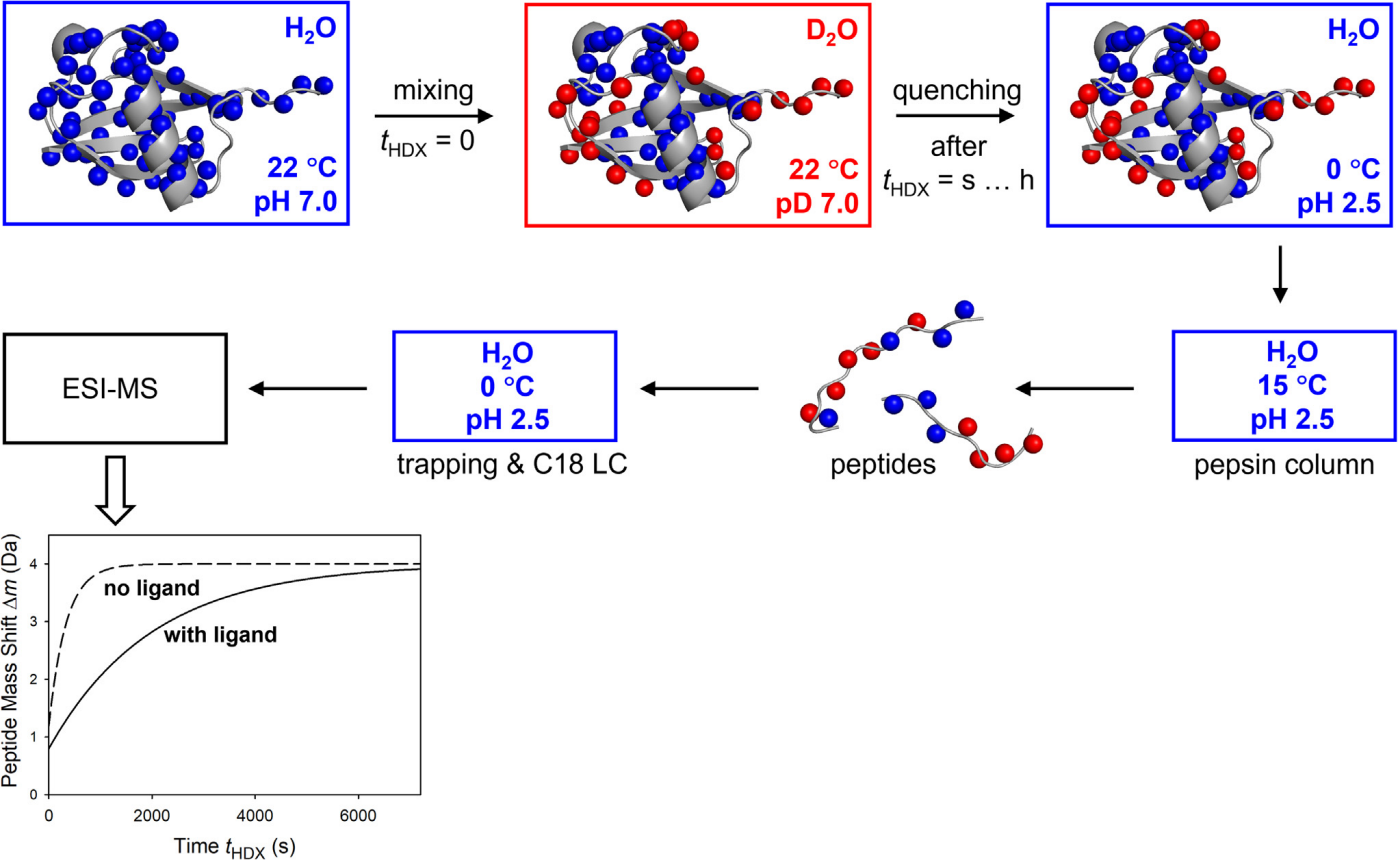
**Schematic depiction of the standard bottom-up HDX-MS workflow.** The *red box* refers to a D_2_O environment. *Blue boxes* indicate steps that take place in H_2_O. *Blue spheres* represent hydrogen (^1^H) and *red spheres* represent deuterium (^2^H). The locations of these spheres represent NH_j_ sites in ubiquitin. The *bottom-left panel* schematically indicates the behavior of a peptide that exhibits enhanced protection in the presence of a ligand that binds to the protein.

Except for the incorporation of automation, robotics, and enhanced data analysis tools (26, 90, 91, 92, 93), HDX-MS experiments have remained essentially unchanged over the past 30 years (Fig. 3) (23, 24). After exposing the protein to D_2_O labeling buffer under physiological conditions, aliquots are removed at various time points *t*_HDX_. Processing of these aliquots takes place in H_2_O solution, causing a certain level of ND → NH back exchange (discussed in more detail below). To promote deuterium retention, the aliquots are quenched by acidification to pH 2.5. In addition, the post-quenching steps are conducted at low temperatures (59). Protein digestion takes place on a column that contains an immobilized acidic protease, most commonly pepsin (94, 95, 96, 97, 98). The resulting peptides are captured on a trapping column, followed by reverse-phase liquid chromatography (LC) and online electrospray ionization (ESI) MS (90, 99, 100). This workflow is outlined in Figure 3; see (101) for a more detailed description of the flow path in a typical commercial HDX system.

The readout of HDX-MS data involves tracking the mass *m* of each peptide (averaged over all isotope peaks) as a function of *t*_HDX_, keeping in mind that each peptide reports on the HDX behavior of a certain protein region. The peptide nominal mass shift Δ*m*(*t*_HDX_) = *m*(*t*_HDX_) – *m*_H2O_ equals the number of deuterium atoms in the peptide, with *m*_H2O_ being the mass of the non-deuterated peptide in water. The bottom panel of Figure 3 schematically illustrates a scenario where a peptide shows enhanced protection in the presence of a ligand, indicating that ligand binding stabilizes the corresponding protein region.

Various modifications of the workflow in Figure 3 have been implemented, including the use of MALDI (matrix-assisted laser desorption/ionization) instead of ESI (85, 102), rapid mixing and pulse-labeling (32, 91, 103, 104), gas phase fragmentation (32, 105), as well as ion mobility spectrometry (106). Instead of labeling an initially non-deuterated protein in D_2_O, it is also possible to perform the experiment in reverse, *i.e.* label a fully deuterated protein in H_2_O. The latter approach can be advantageous under certain conditions (107).

## “Can We Get Serious Now?”

Although this Tom Hanks quote from the movie ”Sully” has nothing to do with proteins, it fittingly captures a certain disconnect that currently afflicts the HDX-MS arena: The concepts expressed in Equations (1), (2), (3), (4) have been repeated countless times in the HDX-MS literature, inadvertently promoting the misconception that it is possible to measure *k*_HDX_j_ and *P*_j_ values by HDX-MS. In reality, typical HDX-MS data only provide *qualitative* information about *changes* in protection patterns with *moderate* structural resolution. Each of these aspects represents a limitation, as explained below.

(i) Instead of probing individual NH_j_ sites, the spatial resolution of HDX-MS is typically around 5 to 20 residues, as determined by the length of proteolytic peptides. The only technique that can routinely measure single residue *k*_HDX_j_ values is 2D-NMR spectroscopy, based on monitoring the exp(-*k*_HDX_j_ *t*_HDX_) signal decay of individual N^1^H resonances (29, 30, 31). There have been various attempts to increase HDX-MS spatial resolution toward the single-residue level (108), including electron-based fragmentation (33, 105, 109, 110, 111) and the use of subtractive methods involving partially overlapping peptides (103, 112, 113, 114). However, those approaches are not widely used because they are either cumbersome, difficult to validate, not supported on commercial platforms, or prone to artifacts (115).
(ii) Unlike *P*_j_ values (Equation 4), which are a robust descriptor of amide backbone behavior, HDX-MS does not generally provide truly quantitative information. Instead, HDX-MS analyses often fit peptide deuteration kinetics to exponential functions using two or three “apparent” rate constants that do not have direct physical meaning, other than providing a phenomenological fit of the data (116).
(iii) Rather than delivering an absolute readout of protein dynamics, HDX-MS usually requires experiments to be conducted in comparative fashion, by interrogating a protein under two (or more) conditions. One of these conditions serves as reference, allowing conclusions such as “residues X to Y become less dynamic after protein exposure to condition Z”. Examples of such comparative measurements include the identification of regions that show altered dynamics before/after ligand binding (16, 24, 117), probing mechanical stress under molecular motor on/off conditions (118), uncovering allosteric signal transmission upon effector addition/removal (11, 18, 119), and epitope mapping *via* experiments with/without antibodies (120, 121).

## Amide Back Exchange—An Inconvenient Reality

A complication of bottom-up HDX-MS is the fact that ND sites gradually revert to NH after quenching. Low levels of amide back exchange are desirable for various reasons, *e.g.*, they enhance the dynamic range of the HDX-MS workflow by increasing peptide mass shifts. Also, there are many scenarios where experimentalists strive to determine the absolute number of exchanged hydrogens in a peptide as a function of *t*_HDX_ (114, 122). This is particularly important for approaches aimed at using HDX-MS data for developing atomistic models of protein structure and dynamics (66, 67, 68, 69, 70, 71, 72, 73, 74), and for efforts to improve HDX-MS spatial resolution *via* overlapping peptides (103, 108, 112, 113, 114, 115, 123).

Back exchange is of relatively little concern for comparative HDX-MS experiments aimed at detecting qualitative changes in protein behavior under different conditions (*e.g.*, with/without ligand, see above) (16, 24, 117). Relative back exchange levels for any peptide in such paired samples are the same, therefore, trends such as enhanced or reduced deuteration in certain segments can be detected without accounting for back exchange (124). Many studies have successfully used this approach for examining protein dynamics in a qualitative fashion. However, any attempts to extract quantitative details from HDX-MS data must pay close attention to back exchange.

Back exchange occurs because protease column digestion, peptide trapping, and LC separation expose ND sites to H_2_O (Fig. 3) (115). Quench conditions (pH 2.5, ∼0 °C) are maintained as much as possible to reduce back exchange, taking advantage of the fact that *k*_ch_j_ values are at a minimum under these conditions (59). To minimize back exchange further, post-quenching steps are performed as quickly as possible, typically in <20 min (90, 100). In addition to bulk solution-phase back exchange (26), gaseous peptide ions undergo gas phase back exchange while they interact with H_2_O vapor in the ion sampling interface of the ESI mass spectrometer (22, 90, 125). Back exchange may also take place while peptides reside within ESI droplets (126, 127). Overall, solution and gas phase events cause peptides to lose a substantial fraction of their backbone NH deuteration, often around 30% but sometimes 50% or more for some peptides (26, 128, 129, 130).

Deuteration of OH, NH, and SH sites in side chains is completely lost due to back exchange during the workflow of Figure 3, with the possible exception of Arg NδH (59, 100). Near-complete side chain back exchange is not a disadvantage; on the contrary, it simplifies the readout by allowing experimentalists to focus on backbone sites. Most carbon-linked hydrogens are non-exchangeable. Only the C2H position of His side chains can retain some deuterium, but this is insignificant under typical conditions because deuteration at this site is very slow (124, 131, 132).

Many experiments require compromises, in the form of higher temperatures or longer processing times, even if this means increased back exchange. For example, commercial HDX-MS systems perform digestion at around 15 °C to boost the peptide yield, before cooling the solution back down to 0 °C for trapping and LC (90). Also, extensions of the Figure 3 standard methodology can prolong the back exchange period, such as disulfide reduction (133, 134), deglycosylation (135, 136), size exclusion (137), cation exchange (138), or tag-and-capture steps (139). In each of these cases, the workflow has to be carefully optimized to ensure that the back exchange remains within acceptable limits.

Novices sometimes propose to suppress back exchange by using a D_2_O-based mobile phase instead of H_2_O. However, this strategy is equally detrimental because it causes the opposite problem, *i.e.*, the forward exchange of residual NH sites to ND. Thus, regardless of the isotope makeup of the mobile phase, the distribution of NH and ND sites imprinted onto the protein during *t*_HDX_ is gradually washed away during the digestion/LC/ESI-MS workflow.

## Back Exchange Modeling

Being able to conduct back-exchange predictions is important for optimizing HDX-MS workflows. Despite pioneering work by Zhang and Smith in the early 1990s (23), such concepts have received only little attention in the more recent literature (100, 113), prompting us to briefly summarize some pertinent points. The *D*_j_ contribution of each residue to the peptide deuterium content depends on the labeling period *t*_HDX_ and the back exchange period *t*_BX_. In the simplest case, back exchange proceeds with a single rate constant *k*_BX_j_ that may be approximated by *k*_ch_j_ under quench conditions, such that the deuterium content at amide site *j* is

(5) $$D_{j}\left(t_{HDX},t_{BX}\right)=\left(1-\exp\left[-k_{HDX\_j}{\;t}_{HDX}\right]\right)\times \exp\left[-k_{BX\_j}\;t_{BX}\right]$$

More elaborate descriptions will incorporate the fact that back exchange usually takes place in distinct stages, each of which has its own *k*_BX_j_ and *t*_BX_. In typical HDX systems (90) rapid back exchange initially takes place during digestion at ∼15 °C, followed by slower deuterium loss during peptide trapping and LC at 0 °C. These two stages can be accounted for by multiplying the right-hand side of Equation 5 by the appropriate exp [-*k*_BX_j_ *t*_BX_] terms. Back exchange in ESI droplets and in the gas phase represents the final stage that can be considered in a similar fashion.

**Fig. 4:**
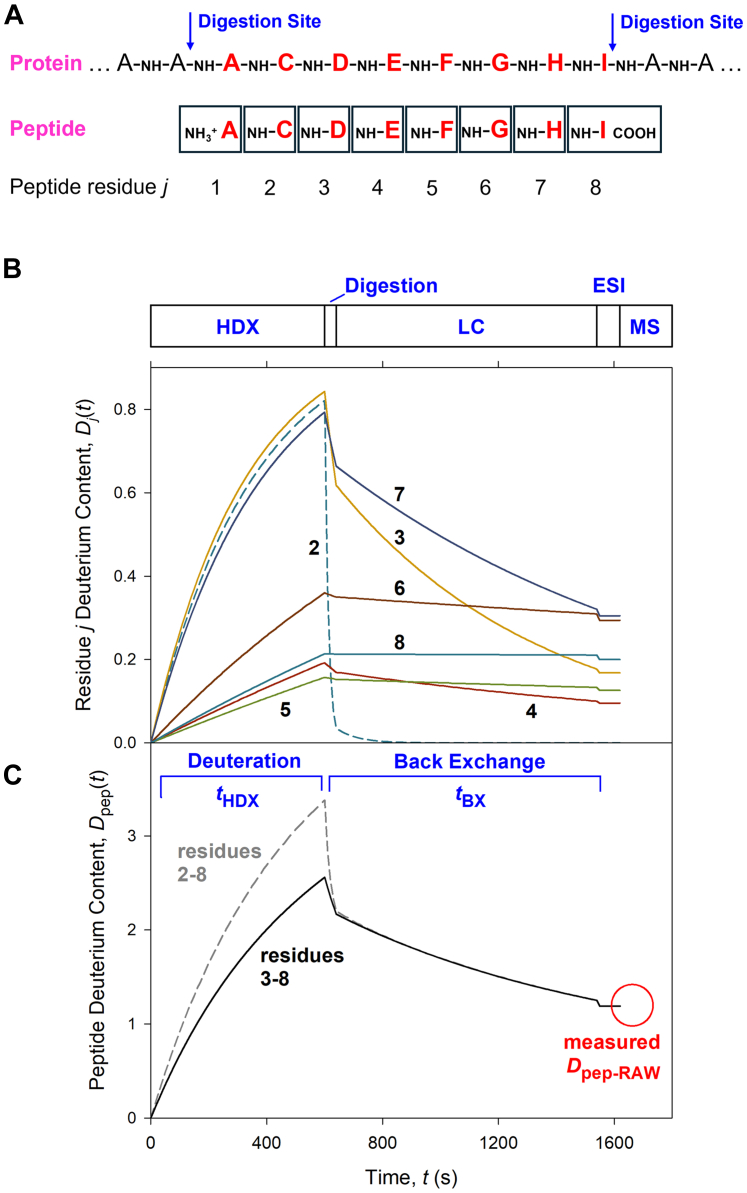
**Kinetics of protein backbone deuteration and peptide back exchange during bottom-up HDX-MS.***A*, fictitious stretch of protein sequence, producing the peptide ACDEFGHI after digestion. *B*, deuterium content of each NH site *j* vs. time, with *k*_ch_j_ values from refs. (59, 78). (i) 10 min HDX in D_2_O under native conditions at pD 7.4, 22 °C, with log *P*_j_ = 4 for all sites. (ii) 40 s pepsin digestion at 15 °C, pH 2.5 in H_2_O. (iii) 15 min peptide LC at 0 °C, pH 2.5 in H_2_O. (iv) Peptide ESI-MS with 5% droplet/gas phase back exchange. *C*, Peptide deuterium content *D*_pep_(*t*) calculated from panel B for residues 2 to 8 and 3 to 8. ESI-MS analysis takes place much faster than indicated here.

Using the aforementioned strategy, Figure 4 depicts Excel-generated data of protein deuteration and back exchange under typical HDX-MS conditions (90). For illustrative purposes, we arbitrarily chose the alphabetical peptide sequence ACDEFGHI (Fig. 4*A*). Following native protein deuteration for *t*_HDX_ = 10 min at pD 7.4, back exchange at pH 2.5 was modeled in three stages (Fig. 4*B*). (i) An initial rapid burst of deuterium loss takes place during the 40 s of peptic digestion at 15 °C. For simplicity, our calculations neglected forward and back exchange during the brief interval between quenching and the onset of digestion. Also, it was assumed that digestion takes place completely in H_2_O, even though some D_2_O is initially present during this step, prior to being replaced by the LC mobile phase. (ii) Back exchange during the subsequent peptide trapping and LC at 0 °C proceeds more slowly, but this second stage is responsible for most of the deuterium loss due to the longer time window, *i.e.*, 15 min in this example. (iii) Droplet and gas phase back exchange during ESI was modeled as an additional 5% deuterium loss prior to mass analysis, *via* multiplication of all *D*_j_ values by 0.95 (22, 90, 125).

Figure 4*C* shows the deuterium content of the peptide, *D*_pep_(*t*), obtained by summation of all *D*_j_(*t*) contributions. Note that *t* in this expression represents the time of the entire workflow, from the start of deuteration up until ion detection. Residue 1 is not included in the summation because digestion converts the amide on residue 1 into an amine that undergoes complete back exchange, similar to side chain sites (59, 100). Residue 2 exhibits very rapid back exchange as well, such that HDX-MS can only monitor deuteration from residue 3 onward (black profile in Fig. 4*C* and Equation 6). Quasi-complete deuterium loss at the first two residues is common to most peptides (59), and it represents an important consideration when assessing the spatial resolution of an HDX-MS experiment. Partial deuterium retention at residue 2 is possible only if the first two residues carry side chains that are bulky and hydrophobic (W, L, V, I) (100). Except for these rare cases, *D*_pep_(*t*) for a peptide consisting of *N* amino acids is composed of individual *D*_j_(*t*) contributions according to

(6) $$D_{\text{pep}}\left(t\right)=\sum_{i=3}^{N}D_{j}\left(t\right)$$

with summation starting at residue 3 rather than 2. Thus, the maximum attainable *D*_pep_ is *D*_pep_max_IDEAL_ = *N*–2 (26).

The back exchange level (%BX) of an HDX-MS experiment can be determined by monitoring how much peptide deuterium is detected after exposing a fully deuterated protein (124) to the workflow of Figure 3, starting at the quenching step. If the raw data of a so-called *m*_100_ sample reveal an experimentally measured deuteration level *D*_pep_max_RAW_, the back exchange level is %BX = 1 - *D*_pep_max_RAW_/*D*_pep_max_IDEAL_. For the scenario in Figure 4, *D*_pep_max_RAW_ = 3.6 and *D*_pep_max_IDEAL_ = 6, corresponding to %BX = (1–3.6/6) = 40%. For simplicity, these calculations neglect forward exchange; see ref. (26) for more details.

Back exchange can be suppressed by shortening the workflow after deuteration. For the scenario considered in Figure 4, reducing the digestion time (40 s → 20 s) and speeding up the LC gradient (15 min → 7.5 min) would result in %BX = (1–4.4/6) = 27%. However, a reduced digestion time will likely lower the peptide yield and sequence coverage. Similarly, the faster LC gradient may compromise the quality of peptide separation. All of these considerations have to be factored in when optimizing HDX-MS workflows.

## Correcting for Back Exchange—An Unsolved Problem

As indicated in Figure 4*C*, the experimentally detectable deuteration levels of each peptide (the “raw” data) consist of individual residue contributions according to

(7) $$D_{\text{pep}-\text{RAW}}=\sum_{i=3}^{N}D_{i}\left(t_{HDX},t_{BX}\right)$$

An ideal HDX-MS experiment would measure deuteration levels prior to back exchange, *i.e.*, at the peak of the black *D*_pep_ profile in Figure 4*C*. Taking for granted that complete back exchange of residue 2 is inevitable, we define this ideal deuteration profile as

(8) $$D_{\text{pep}-\text{IDEAL}}=\sum_{i=3}^{N}D_{i}\left(t_{HDX},0\right)$$

It is desirable to transform experimentally measured *D*_pep_RAW_ data into corrected data *D*_pep_CORR_ that match D_pep_IDEAL_ as closely as possible. A correction strategy was proposed more than 30 years ago (Equation 9) (23), and this strategy has been widely used ever since (24, 26, 27, 49, 50, 123).

(9) $$D_{\text{pep}-\text{CORR}}\left(t_{HDX}\right)=\frac{m\left(t_{HDX}\right)-m_{0}}{m_{100}-m_{0}}\times \left(N-2\right)$$

Here, *m*(*t*_HDX_) is the measured peptide mass (representing *D*_pep_RAW_ in Fig. 4*C*), while *m*_100_ and *m*_*0*_ correspond to the measured mass values of fully and minimally deuterated controls, respectively. Despite the widespread use of Equation 9, there have been hardly any efforts to assess the reliability of this correction strategy (23). Simple tests reveal that the performance of Equation 9 is surprisingly poor under some conditions, as illustrated below.

For simplicity, we assume that each *D*_j_ value can be modeled using Equation 5 (with a single *k*_BX_j_), and that artifactual forward exchange is negligible (*m*_0_ ≈ *m*_H2O_). When using the previously introduced notation, Equation 9 becomes

(10) $$D_{\text{pep}-\text{CORR}}\left(t_{\mathrm{HDX}}\right)=\frac{D_{\text{pep}-\text{RAW}}}{{{D_{\text{pep}}}_{\_\max}}_{\_\text{RAW}}}\times \left(N-2\right)$$

Let us first consider a peptic tripeptide that possesses only a single observable deuteration site on residue 3. In this case, Equations 9 and 10 transform into

(11) $$D_{\text{pep}-\text{CORR}}\left(t_{HDX}\right)=\frac{\left(1-\exp\left[-k_{HDX}{\;t}_{HDX}\right]\right)\times \exp\left[-k_{BX}{\;t}_{BX}\right]}{\exp\left[-k_{BX}{\;t}_{BX}\right]}\times \left(N-2\right)$$

where the $\exp\left[-k_{BX}{\;t}_{BX}\right]$ back exchange term in numerator and denominator cancels out, such that *D*_pep_CORR_ = *D*_pep_IDEAL_. In other words, for peptides that only contain a single observable deuteration site, the Equation 9 correction strategy works perfectly.

**Fig. 5:**
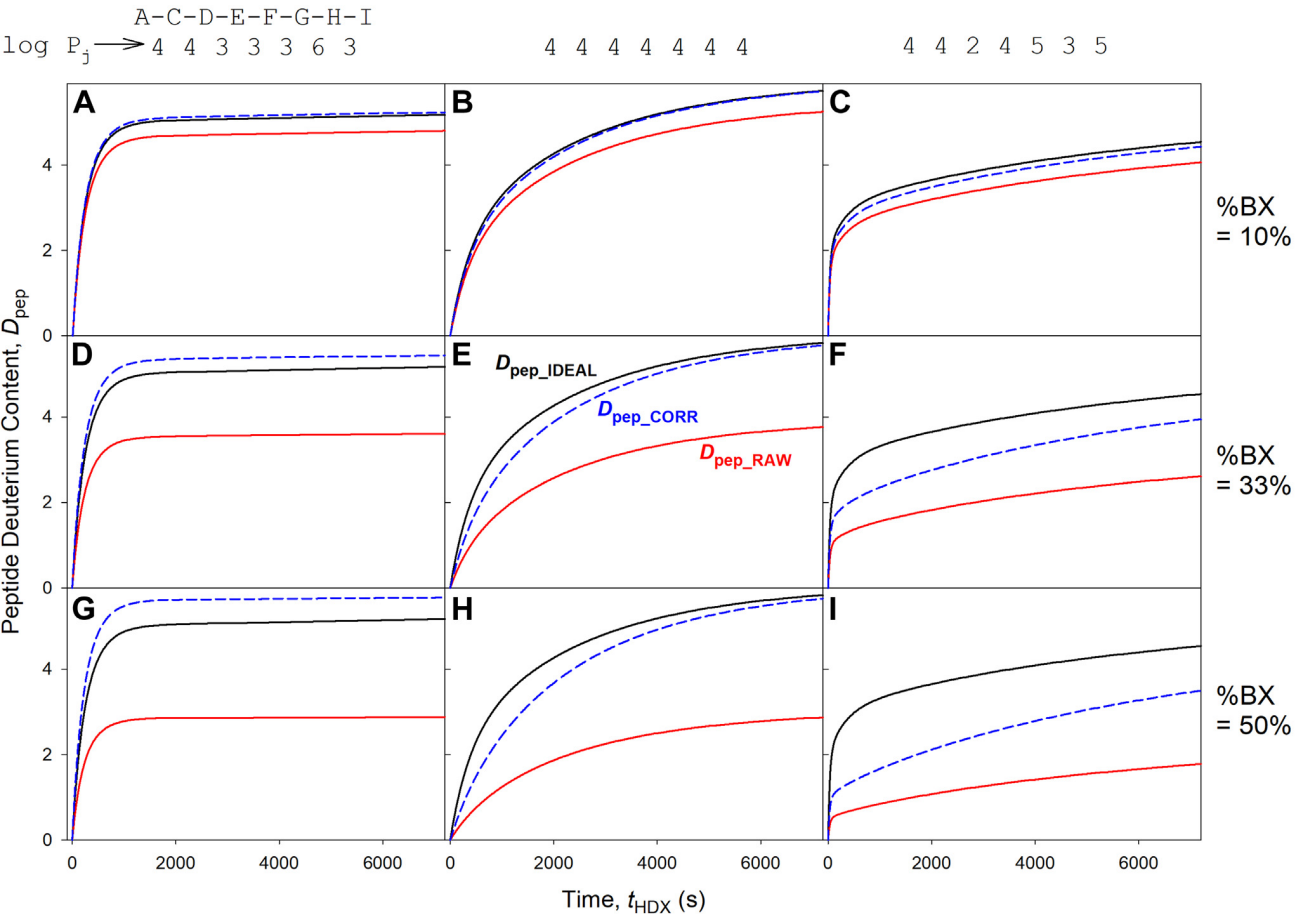
**Performance of the**Equation 9**back exchange correction for the model peptide ACDEFGHI.** Each panel shows three HDX profiles, calculated as discussed in the text: *D*_pep_IDEAL,_ ideal scenario without back exchange for residues 3 to 8; *D*_pep_RAW_, experimentally measurable raw data; *D*_pep_CORR_, raw data after back exchange correction (Equations 9 and 10). *A*–*C*, 10% back exchange, *t*_BX_ = 200 s; (*D*–*F*) 33% back exchange, *t*_BX_ = 1000 s; (*G*–*I*) 50% back exchange, *t*_BX_ = 2000 s. Each column shows data for one set of log *P*_j_ values, listed along the top.

Unfortunately, the observation of tripeptides in experimental HDX-MS data is uncommon, as typical proteolytic peptides are much longer (short peptides tend to elute together with salt contaminants close to the solvent front, rendering them undetectable). How does the correction method of Equation 9 fare for longer peptides? We examine this question by returning to the ACDEFGHI model peptide introduced earlier. Each panel in Figure 5 depicts three deuteration profiles for this peptide. *D*_pep_IDEAL_ profiles (black) are shown for three sets of protection factors, listed as log *P*_j_ along the top. Experimentally measurable *D*_pep_RAW_ data are shown in red; back exchange implies that *D*_pep_RAW_ < *D*_pep_IDEAL_ for all time points. Finally, *D*_pep_CORR_ profiles (blue dashed lines) were calculated by applying the Equation 9 correction strategy to the *D*_pep_RAW_ data. Each row in Figure 5 corresponds to a specific back exchange level, as noted along the right-hand side. A glance at Figure 5 reveals that none of the *D*_pep_CORR_ profiles coincides with the corresponding *D*_pep_IDEAL_ data, although the severity of the deviation depends on the conditions. The reason for these discrepancies is that for *N* > 3 both the numerator and denominator in Equation 10 are sums of (*N*-2) *D*_j_ elements, each with its unique exp(-*k*_BX_j_ *t*_BX_) (Equation 5). Unlike in Equation 11, these exp(-*k*_BX_j_ *t*_BX_) terms do not cancel out because *k*_BX_j_ for different residue types can differ by more than one order of magnitude (59, 78). Thus, the Equation 9 correction works perfectly only for single-residue exchange (*N* = 3, as in Equation 11) or for poly-X peptides with only one type of amino acid.

For any given peptide sequence, the magnitude of the error associated with the Equation 9 correction depends on the protection factors *P*_j_ and on %BX. For %BX = 10%, *D*_pep_CORR_ and *D*_pep_IDEAL_ profiles are quite close to one another. Unfortunately, such low %BX values are not easily attainable in bottom-up experiments, where typical values are around 30% (Fig. 5, *D*–*F*) and up to 50% for some peptides (Fig. 5, *G*–*I*) (26, 128, 129, 130). For the scenarios of Fig. 5, *F* and *I*, the Equation 9 correction fails, generating *D*_pep_CORR_ profiles that are dramatically different from *D*_pep_IDEAL_. In general, the Equation 9 correction strategy is likely to produce large errors when %BX is high (≥30%), and when *k*_ch_j_ values in the peptide of interest are very heterogeneous.

The poor performance of the Equation 9 correction method under some of the test conditions in Figure 5 is disconcerting, considering that much of the field has promoted this strategy for many years (24, 26, 27, 49, 50, 123). This issue is of particular concern for ongoing efforts to use HDX-MS data as constraints for atomistically modeling protein structure and dynamics (66, 67, 68, 69, 70, 71, 72, 73, 74, 75, 76). Such efforts rely on the comparison of model-generated HDX profiles with *D*_pep_CORR_ data. Evidently, any attempt to steer a protein dynamics model toward the distorted dashed *D*_pep_CORR_ profiles in Fig. 5, *F* and *I* instead of the proper *D*_pep_IDEAL_ could introduce major errors into the model predictions.

## Strategies for Tackling the Back Exchange Problem

How can the back exchange problem be solved? One possibility is the development of improved correction strategies. However, any method that relies on calculated *k*_ch_j_ ≈ *k*_BX_j_ values will face uncertainties, because *k*_ch_j_ data may not properly capture peptide conformational effects and peptide-column interactions (115). Also, strategies involving *m*_100_ samples have to deal with the fact that the production of fully deuterated controls is non-trivial (124). Machine learning could offer interesting opportunities (122), but generating adequate training data for such strategies is challenging, and validation concerns will have to be addressed.

Another possibility is to employ experimental methods that provide less back exchange than commonly used bottom-up workflows. Such methods include the careful adjustment of pH and ionic strength during LC (128), the use of subzero Celsius LC (140, 141, 142, 143, 144), aprotic mobile phases (145), or the application of rapid on-line systems where %BX < 10% is achievable (91). Although highly promising, such improved workflows may introduce new uncertainties because one can no longer assume complete back exchange at peptide residue 2; also, partial retention of side chain deuterium may interfere with data analysis (59, 100).

The back exchange problem can also be tackled by employing top-down analyses immediately after HDX, bypassing solution phase digestion and separation. Electron-based fragmentation of electrosprayed protein ions (electron capture/transfer dissociation, ECD/ETD) (146, 147) is promising in this context, sometimes providing spatial resolution close to the single residue level (33, 105, 109, 111, 148). However, top-down approaches have not yet been widely adopted by the HDX community. One aspect that makes top-down HDX challenging is the requirement for very gentle source conditions, because collisional heating causes scrambling, *i.e.*, rapid H/D migration that randomizes spatial labeling patterns (105). Also, ECD/ETD fragmentation efficiencies tend to be quite low, necessitating extensive signal averaging. Although HDX time profiles are obtainable under such conditions (111), most top-down studies have focused on single-time point measurements.

Another interesting idea is the application of ECD or ETD for peptide fragmentation in bottom-up experiments, an approach that can provide single-residue resolved HDX data (105) when using properly optimized low-scrambling source conditions (149). A strategy analogous to Equation 11 should be suitable for the back exchange correction of such single-residue bottom-up data.

In summary, a silver bullet for overcoming the challenges associated with back exchange has yet to be found, calling for renewed efforts to tackle this issue through the development of more robust experimental and data analysis strategies.

## Conclusion

Classical bottom-up HDX-MS workflows probe changes in protein dynamics in response to certain stimuli, such as the addition of noncovalent ligands. Sadly, the qualitative information currently obtainable from such data remains somewhat vague, revealing regions that become “more protected” or “less protected”. It is hoped that future developments will permit the interpretation of HDX-MS data in a more meaningful, quantitative, and atomistic fashion, uncovering the exact range of motions performed by the protein under a given set of conditions. Such enhanced readout strategies require several advances. At the fundamental level, it is essential to understand in more detail what types of dynamic events allow NH deuteration to take place. In other words, it is necessary to develop a better structural understanding of open and closed states in the Linderstrøm-Lang model, or perhaps one should even abandon this simple model in favor of a more advanced framework. Complementing HDX-MS with MD simulations and other simulation tools (80, 82, 150, 151) is a promising strategy for obtaining enhanced insights into the nature of protein dynamic events. Machine learning approaches might be interesting as well (122, 152). At the experimental level, the development of robust workflows with suppressed back exchange will be essential, keeping in mind that practitioners probably put too much faith in the standard correction method (Equation 9). Until HDX-MS can routinely provide deuteration kinetics with single-residue resolution, practitioners should consider complementing their experiments with HDX-NMR data for a detailed characterization of individual amides. Top-down (33, 109, 111, 148) and bottom-up (105) HDX-MS workflows involving ECD or ETD with low-scrambling source conditions (149) are viable strategy for single-residue measurements. Hopefully, such electron-based dissociation techniques will soon find more widespread acceptance in the HDX-MS arena.

Recent years have witnessed the application of HDX-MS to biological systems of ever-increasing size and complexity, and we applaud these efforts. However, at times it is somewhat disconcerting when HDX-MS is used to develop highly intricate biological ideas when there are still so many unknowns associated with the interpretation of HDX-MS data. Although perhaps not very glamorous, there remains an urgent need to develop an improved understanding of HDX fundamentals by focusing on small and simple model systems. It is hoped that the current article will help stimulate work in this direction.

## Data availability

All study data are included in the article and SI Appendix.

## Conflict of interest

The authors declare that they have no conflicts of interest with the contents of this article.
